# Application of Bilateral Sympathetic Nerve Cryoablation as a Bridge to Transplant for Refractory Ventricular Tachycardia

**DOI:** 10.1016/j.atssr.2026.02.005

**Published:** 2026-02-20

**Authors:** Marilina La Porta, Mathieu Echivard, Stéphane Renaud, Joseph Seitlinger

**Affiliations:** 1 Division of Thoracic Surgery, Istituto di Ricovero e Cura a Carattere Scientifico (IRCCS) Azienda Ospedaliero Universitaria Di Bologna, Bologna, Alma Mater Studiorum, University of Bologna, Bologna, Italy; 2 Division of Thoracic Surgery, University Hospital of Nancy, Nancy, France; 3 Division of Cardiology, University Hospital of Nancy, Nancy, France

## Abstract

This case report describes a medically fragile patient with sustained ventricular arrhythmia who was anticoagulated and thus underwent bilateral video-assisted thoracoscopic sympathetic cryoablation as the least-invasive option. This technique provides a temporary, reversible sympathetic block, reducing procedural risks compared with conventional denervation. The gradual inverse nerve remodeling after cryoablation may account for early arrhythmic recurrences. Overall, this approach shows promise for rhythm control and safety in high-risk patients, but further evaluation is required.

Sustained ventricular arrhythmia is a potentially life-threatening condition and a frequent cause of sudden cardiac death. The cause can be found in genetic defects or structural heart disease, and the role of the autonomic nervous system is widely recognized.^1^^,^^2^

The gold standard in recurrent ventricular arrhythmia is pharmacologic treatment in combination or not with ablation for patients nonresponsive. A surgical approach, such as cardiac sympathetic denervation (CSD), can be considered for uncontrolled patients.^3^, ^4^, ^5^ There are several types of CSD by video-assisted thoracoscopic surgery (VATS) to achieve the interruption of the sympathetic chain.^6^, ^7^, ^8^

Managing the patient presented in this report was particularly challenging because he was on curative-dose anticoagulation and could not be switched due to heparin-induced thrombocytopenia while awaiting cardiac transplantation. In this setting, we report the application of a minimally invasive CSD technique involving nerve cryoablation.

A 63-year-old man with mixed ischemic and arrhythmogenic right ventricular cardiomyopathy was referred after recurrent ventricular arrhythmic storms. His history included obesity, type 2 diabetes, dyslipidemia, and hypertension. A myocardial infarction in 2008 was managed by angioplasty of the middle left anterior descending artery. After a cardiorespiratory arrest in 2016, he was found to have biventricular dysfunction (left ventricular ejection fraction, 0.30-0.35) due to apical and lateral necrosis, and received an implantable cardioverter defibrillator (ICD) for secondary prevention. Anticoagulation with argatroban was started after a left intraventricular thrombus was detected the same year.

Despite ICD support, recurrent ventricular fibrillation episodes from 2017 necessitated the addition of amiodarone. In 2019 and April 2023, the patient experienced further ventricular tachycardia (VT) storms, requiring endocavitary ablation procedures combined with medical therapy. Despite optimal medical management and ICD therapy, he continued to present with multiple VT storms in May, July, and September 2023. Consequently, he underwent 3 endocavitary ablations and 1 combined endocavitary and epicardial ablation, which yielded only partial control of the arrhythmias.

By January 2025, even with optimized cardiotropic and antiarrhythmic (Amiodarone) medications, and after 4 VT ablation procedures, the patient had 3 more episodes of electrical storm. Multidisciplinary consultations among cardiologists, electrophysiologists, and the cardiac transplant team led to his enrollment on the semiemergency cardiac transplantation list. Given the persistent VT and in anticipation of transplantation, bilateral sympathectomy surgery was indicated and validated.

On March 15, 2025, the patient underwent bilateral VATS for sympathectomy using cryoablation of the sympathetic nerve chain. Under general anesthesia with a double-lumen endotracheal tube, he was positioned supine, half-seated, with arms abducted to 90° for optimal axillary exposure (Figure 1). During induction, he experienced severe hypotension requiring vasopressor support and a brief episode of asystole, which resolved spontaneously. One-lung ventilation was established, and 3 ports were placed: in the third intercostal space on the axillary line, and in the fifth intercostal space on both the anterior axillary line and middle clavicular line.

**Figure 1:**
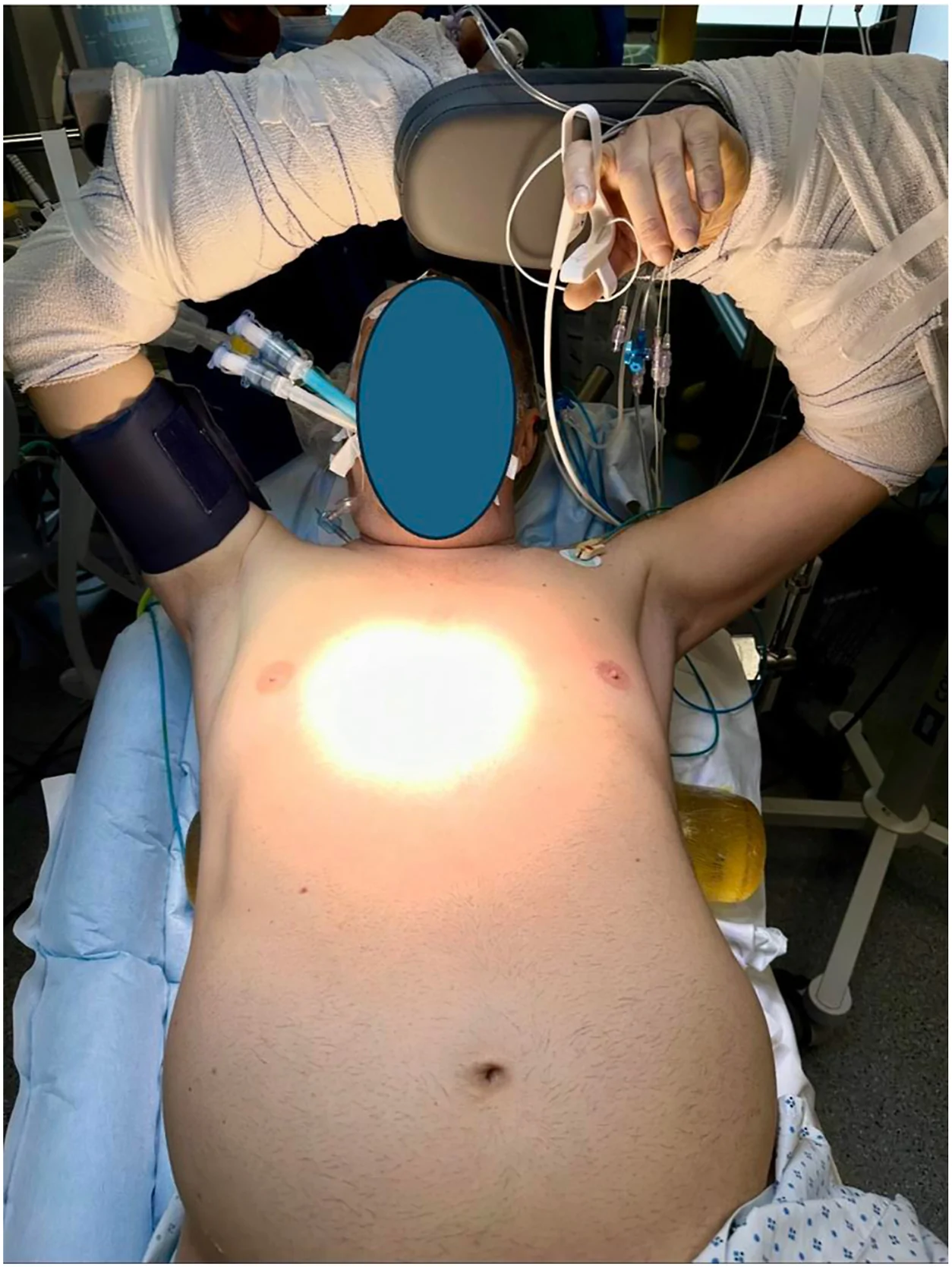
The sequential video-assisted thoracoscopic surgery procedure was performed with the patient supine with arms abducted at 90°.

Owing to the predominance of left-sided sympathetic cardiac innervation, the procedure commenced on the left, which was technically challenging because of the descending aorta and necessitated suction for exposure. A 5-mm 30° camera was used to identify the sympathetic chain and stellate ganglion beneath the pleura. Intercostal spaces were marked, and the cryoSPHERE (AtriCure) cryo nerve block probe was applied to the nerve at the second intercostal space, freezing at −70°C for 120 seconds, followed by active defrosting. This was repeated for the third and fourth intercostal space, and then mirrored on the right side (Figures 2 and 3).

**Figure 2:**
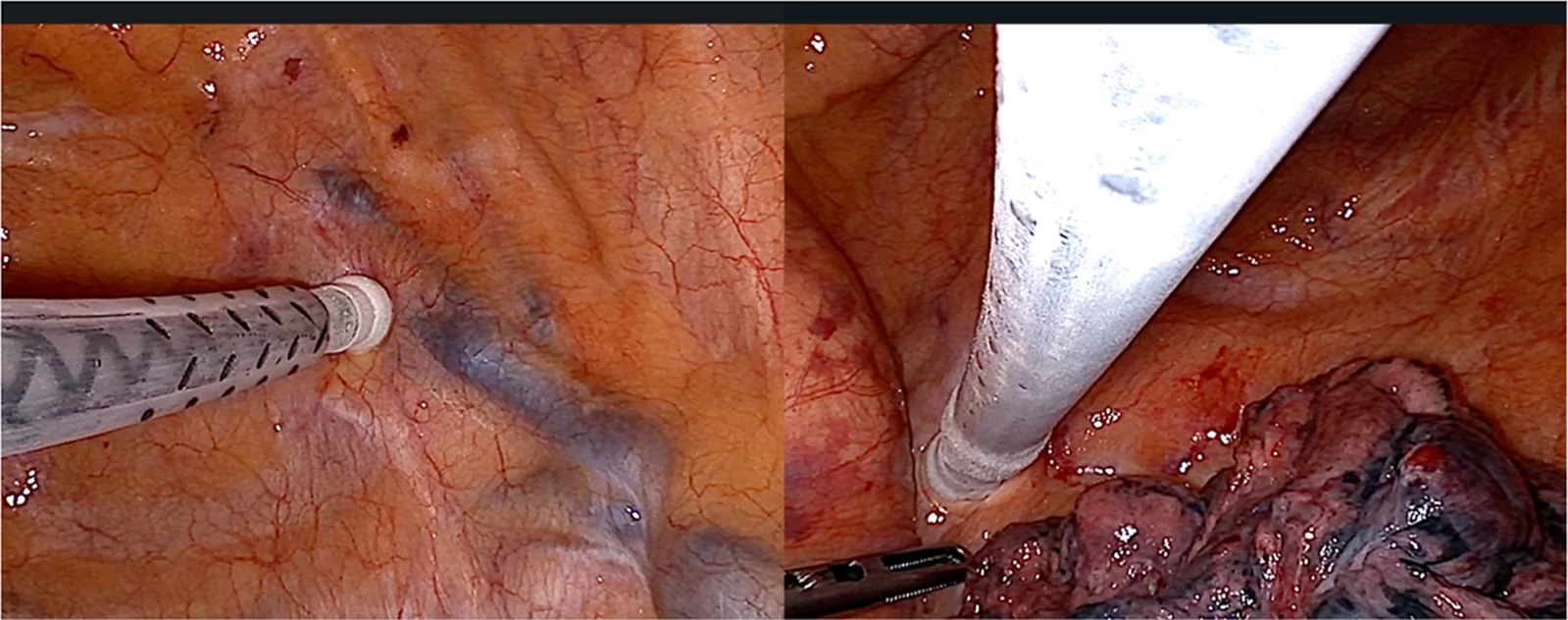
Cryoablation of sympathetic chain with nerve block probe on the left and right side.

**Figure 3:**
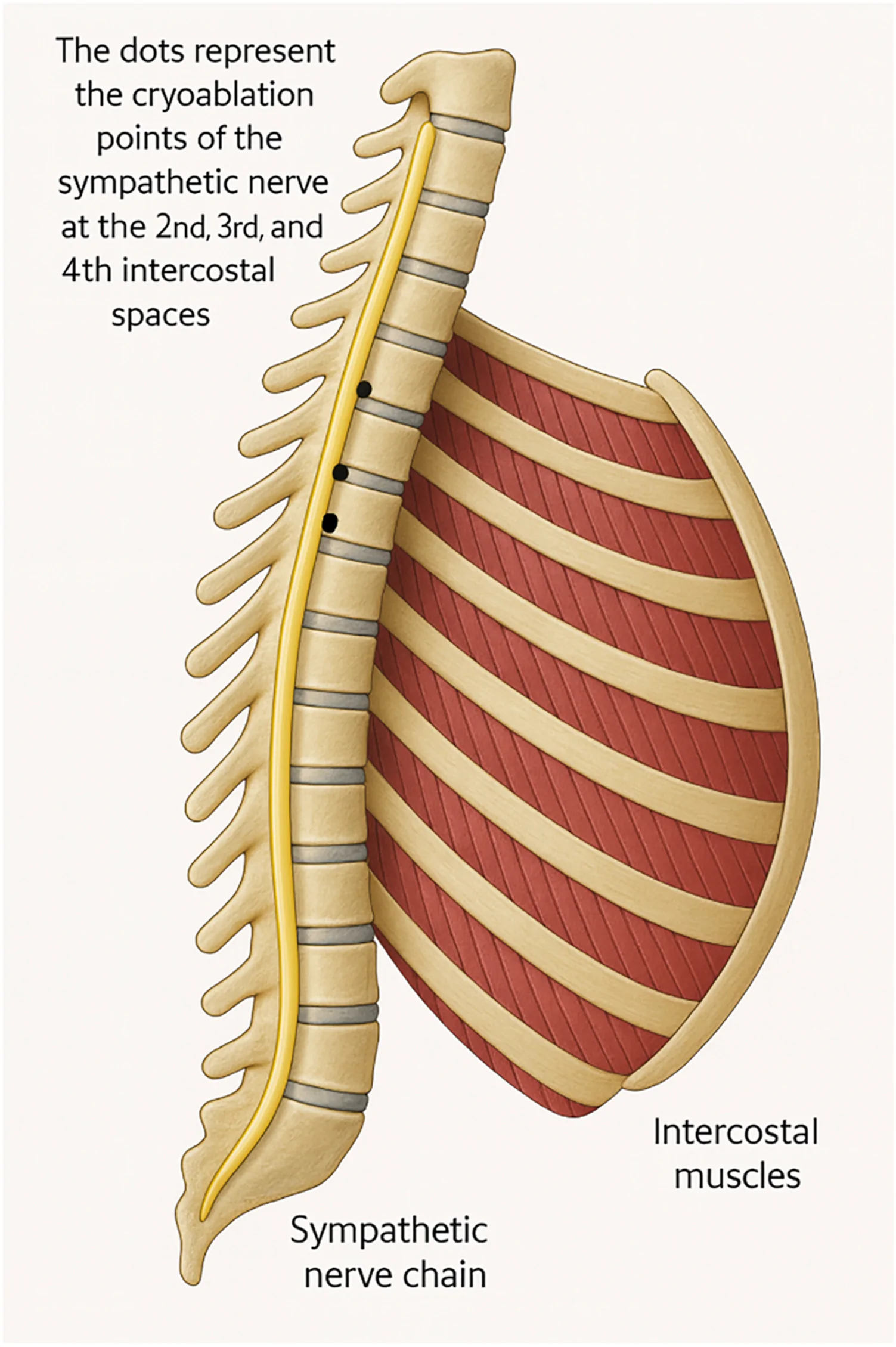
Diagram illustrates the rib cage, spine, and intercostal spaces, with representation of the cryotherapy levels performed (second, third, and fourth intercostal spaces).

A 24F silicone drain was placed on the right side and removed on postoperative day 1 after chest roentgenogram confirmed full lung expansion. The patient was monitored in the cardiology intensive care unit for 48 hours postoperatively. Telemetric surveillance revealed no recurrence of ventricular arrhythmias, and he was transferred to the cardiology unit for further care. His hospital course was uneventful, and he was discharged home on March 24, 2025. after a favorable recovery.

In the 10 days after surgery, no arrhythmic recurrences were observed. Over the subsequent 5 months, the patient experienced 18 VT episodes, including 1 arrhythmic storm and no ICD shocks, compared with 24 VT episodes, 3 arrhythmic storms, and 2 ICD shocks in the 2 months before surgery. Notably, from June through October 2025, follow-up revealed no new arrhythmic events, indicating a marked improvement in rhythm control postprocedure.

## Comment

The autonomic nervous system regulates heart rate, rhythm, conduction, and arrhythmia susceptibility. Research shows that sympathetic overactivation can promote refractory VT in patients with channelopathies and ischemic and nonischemic cardiomyopathies.^1^, ^2^, ^3^, ^4^

CSD is a recognized treatment for refractory VT, particularly during electrical storms or when standard therapies fail.^3^, ^4^, ^5^ Classical CSD, often performed through resection of the thoracic sympathetic chain, results in a significant reduction in defibrillator shocks and an improvement in survival without shock or transplantation at 1 year.^6^

Nonetheless, this technique presents risks, such as intraoperative bleeding and complications from partial stellate ganglia resection, including Claude Bernard-Horner syndrome and irreversible nerve damage.

Given these drawbacks, bilateral sympathetic cryoablation through a VATS approach offers an innovative alternative, particularly as a bridge to transplant. Its main advantage is the temporary and reversible sympathetic block achieved by nerve freezing, typically lasting 3 to 6 months. This reduces the risk of permanent complications and intraoperative bleeding, while maintaining nerve structure and allowing potential functional recovery after transplantation.

This temporary block benefits transplant candidates by enabling postoperative autonomic recovery. In contrast to permanent denervation, cryoablation allows a controlled return of sympathetic function. Nonetheless, uncertainties remain regarding the duration of the block, long-term antiarrhythmic efficacy, and risks linked to the gradual restoration of sympathetic tone after transplantation.

Our initial experience in a bridge to transplant context demonstrated a significant reduction in arrhythmic episodes and improved rhythm control, with enhanced operative safety. Although the technique appeared effective in the present case, we emphasize that transient sympathetic cryoablation using VATS ought to remain reserved for carefully selected patients—specifically those in whom medical therapy has proven ineffective and who present risk factors for surgical complications, such as anticoagulation in this instance. In this setting, the approach emerges as a promising, minimally invasive option for patients awaiting cardiac transplantation when other therapies have failed. Further research is needed to clarify block duration, long-term outcomes, and optimal patient selection, but this technique offers a novel balance of efficacy, safety, and reversibility in a high-risk population.

## References

[1] Zipes D.P. (1970). Cardiac sympathectomy for arrhythmias. N Engl J Med.

[2] Shen M.J., Zipes D.P. (2014). Role of the autonomic nervous system in modulating cardiac arrhythmias. Circ Res.

[3] Téllez L.J., Garzón J.C., Vinck E.E., Castellanos J.D. (2019). Video-assisted thoracoscopic cardiac denervation of refractory ventricular arrhythmias and electrical storms: a single-center series. J Cardiothorac Surg.

[4] Vaseghi M., Gima J., Kanaan C. (2014). Cardiac sympathetic denervation in patients with refractory ventricular arrhythmias or electrical storm: intermediate and long-term follow-up. Heart Rhythm.

[5] Chihara R.K., Chan E.Y., Meisenbach L.M., Kim M.P. (2021). Surgical cardiac sympathetic denervation for ventricular arrhythmias: a systematic review. Methodist Debakey Cardiovasc J.

[6] Gurau A., Bosmans F., Barth A., Brock M.V., Ha J.S. (2024). Exploring cardiac sympathetic denervation as a treatment approach for recurrent ventricular arrhythmias: a concise review. J Clin Exp Cardiolog.

[7] Cauti F.M., Rossi P., Bianchi S. (2021). Outcome of a modified sympathicotomy for cardiac neuromodulation of untreatable ventricular tachycardia. JACC Clin Electrophysiol.

[8] Melinosky K., Leng A., Johnson C.R. (2023). Outcomes comparison of robot-assisted and video-assisted thoracoscopic cardiac sympathetic denervation. Innovations (Phila).

